# Global gene expression profiling data analysis reveals key gene families and biological processes inhibited by Mithramycin in sarcoma cell lines

**DOI:** 10.1016/j.gdata.2014.11.001

**Published:** 2014-11-08

**Authors:** Kirti K. Kulkarni, Kiran Gopinath Bankar, Rohit Nandan Shukla, Chandrima Das, Amrita Banerjee, Dipak Dasgupta, Madavan Vasudevan

**Affiliations:** a Genome Informatics Research Group, Bionivid Technology Pvt Ltd., Bangalore 560043, India; b Biophysics and Structural Genomics Division, Saha Institute of Nuclear Physics, Kolkata 700064, India

**Keywords:** Mithramycin, Sarcoma, Microarray, Global gene expression

## Abstract

The role of Mithramycin as an anticancer drug has been well studied. Sarcoma is a type of cancer arising from cells of mesenchymal origin. Though incidence of sarcoma is not of significant percentage, it becomes vital to understand the role of Mithramycin in controlling tumor progression of sarcoma. In this article, we have analyzed the global gene expression profile changes induced by Mithramycin in two different sarcoma lines from whole genome gene expression profiling microarray data. We have found that the primary mode of action of Mithramycin is by global repression of key cellular processes and gene families like phosphoproteins, kinases, alternative splicing, regulation of transcription, DNA binding, regulation of histone acetylation, negative regulation of gene expression, chromosome organization or chromatin assembly and cytoskeleton.

**Table:** 

Specifications	
GEO accession	GSE25127
Organism	*Homo sapiens*
Cell line	Ewing sarcoma cell lines (TC71 and TC32)
Sex	–
Array type	Expression profiling by array
Platform	GPL570 [HG-U133_Plus_2] Affymetrix human genome U133 plus 2.0 array
Data format	CEL files
Experimental factors	The data consist of 12 arrays. Two cell lines, TC71 and TC32, were treated with solvent control or with Mithramycin, and RNA was extracted at 6 h. Three biological replicates per cell line/treatment
Experimental features	The study aims to define gene expression changes associated with Mithramycin treatment of Ewing sarcoma cell lines
Consent	–
Sample source location	Bethesda, MD — 20892, USA

**Table:** 

Data files				
Accession	Title	Source name	Cell line	Treatment
GSM617274	TC32-M1	TC32 cell line, Mithramycin	TC32	Mithramycin
GSM617275	TC32-M2	TC32 cell line, Mithramycin	TC32	Mithramycin
GSM617276	TC32-M3	TC32 cell line, Mithramycin	TC32	Mithramycin
GSM617277	TC32-S1	TC32 cell line, control	TC32	Control
GSM617278	TC32-S2	TC32 cell line, control	TC32	Control
GSM617279	TC32-S3	TC32 cell line, control	TC32	Control
GSM617280	TC71-M1	TC71 cell line, Mithramycin	TC71	Mithramycin
GSM617281	TC71-M2	TC71 cell line, Mithramycin	TC71	Mithramycin
GSM617282	TC71-M3	TC71 cell line, Mithramycin	TC71	Mithramycin
GSM617283	TC71-C1	TC71 cell line, control	TC71	Control
GSM617284	TC71-C2	TC71 cell line, control	TC71	Control
GSM617285	TC71-C3	TC71 cell line, control	TC71	Control

## Material and methods

Gene expression data for reanalysis was obtained from Gene Expression Omnibus (GEO) database NCBI with the link. http://www.ncbi.nlm.nih.gov/geo/query/acc.cgi?acc=GSE25127. The raw data (CEL file) was normalized and processed using GeneSpring GX V 12.5 (Agilent Technologies Inc., Santa Clara, USA).

### Raw data summarization

All the samples raw data were summarized using the Robust Multi Array Average (RMA) method. RMA is a background correction method that is based on the distribution of Perfect Match (PM) values among probes on an Affymetrix array. It can be used attaching a standard error (SE) to the quantity using a linear model that removes probe-specific affinities [1]. Background corrected, log transformed and Quantile normalized arrays were used and to protect from outliers robust procedures like median Polish are used [2]. Median Polish is an iterative process which operates on a matrix by alternately extracting row and column medians. The convention followed is that the iteration starts with extracting medians for arrays (across probes). Iteration continues until convergence or until a limit on the number of iterations is reached. The limit is of 50 iterations [2].

### Normalization

RMA summarized raw data was Quantile normalized to calculate probe level expression values. Quantile is most widely used pre-processing technique designed to remove technological noise in genomic data. It makes the empirical distribution of all the gene expressions same in the whole experiment [3]. Thus after normalization, all statistical parameters of the sample, i.e., mean, median and percentiles of all samples will be identical. With Quantile normalization (QUANT), a reference array of empirical quantiles, denoted as q = (q_1_,q_2_,…,q_m_), is first computed by taking the average across all ordered arrays. Let y^C^_(1),j ≤_ y^c^_(2),j ≤_ …y^c^_(m),j_ denote the ordered gene expression observations in the jth array (j = 1,2,…,n) of the cth (c = A,B) group, the rth (r = 1,2,…,m) element of this reference array is as follows [3].

$$q_{r}=\frac{1}{2n}\left(\sum_{k=1}^{n}y_{\left(r\right),k}^{A}+\sum_{l=1}^{n}y_{\left(r\right),l}^{B}\right)\text{.}$$

### Baseline transformation

In order to improve the sensitivity of the measurement, baseline transformation of the normalized data is done. This step includes subtraction of an estimated background signal, subtracting the reference signal. Variance ratios were computed for the data set after shifting all measurements upwards by a number of medians for the channel, and subsequently taking the algorithm [4].

### Quality control analysis

Quality control of normalized data is critical to identify inliers and outliers and multiple testing methods are applied for critical evaluation of the data quality.

Box-Whisker plot is a visualization method that requires a sample size of only 5 for analysis [5]. It characterizes a sample using the 25th-lower quartile (Q1), 50th-median (m or Q2) and 75th percentiles-upper quartile (Q3) and the interquartile range (IQR = Q3 − Q1), that covers the central 50% of the data. Quartiles are insensitive to outliers and preserve information about the center and spread. The core element that gives the box plot its name is a box whose length is the IQR and its width is arbitrary [5]. A line inside the box shows the median, which is not necessarily central. Whiskers are conventionally extended to the most extreme data point that is no more than 1.5 × IQR from the edge of the box or all the way to minimum and maximum of the data values.

### Analysis of hybridization controls in the microarray

The hybridization controls show the signal value profiles of the transcripts (only 3′ probe sets are taken) where a line graph is plotted with X axis representing Biotin labeled cRNA transcripts and the Y axis represents the log of the normalized signal values. Typical quality observation is indicated by all samples adhere to the same trend line of internal controls.

### Principal component analysis

(PCA) is a statistical technique for determining the key variables in a multidimensional data set which explains the differences in the observations [6]. PCA is computed by considering the n eigenvalues and their corresponding eigenvectors that are calculated from the n × n covariance matrix of conditions. Each eigenvector defines a principal component. A component can be viewed as a weighted sum of the conditions, where the coefficients of the eigenvectors are the weights. The projection of gene i along the axis defined by the jth principal component is:

$$a\prime_{ij=\sum_{t=1}^{n}a_{{\mathrm{it}\;v}_{tj}}}$$

where v^tj^ is the tth coefficient for the jth principal component; a^it^ is the expression measurement for gene i under the tth condition. A′ is the data in terms of principal components. Since V is an orthonormal matrix, A′ is a rotation of the data from the original space of observations to a new space with principal component axes. The variance for each of the components is associated with its eigenvalue; it is the variance of a component over all probes [6]. Consequently, the eigenvectors with large eigenvalues are the ones that contain most of the information; eigenvectors with small eigenvalues are uninformative.

Correlation-Coefficient analysis reveals the correlation analysis across arrays. It is calculated using Pearson Correlation coefficient as follows:

$$\rho_{X,Y=\;\frac{\;E\left[\left(X-\mu_{X}\right)\left(Y-\mu_{Y}\right)\right]}{\sigma_{X\sigma_{Y}}}}$$

where *σ*_*X*_ is the standard deviation of X, μ_X_ is the mean of X, and E is the expectation.

Condition tree is a hierarchical clustering method where a tree of genes is built by successively finding the two most similar gene expression patterns from the complete data set [7]. It makes use of Distance metric and linkage rule. Distance metric used is Pearson uncentered which is similar to Pearson Correlation coefficient except that the entities are not mean-centered. It is calculated by the following formula

$$\frac{\sum_{i}x_{i}y_{i}}{\sqrt{\sum_{i}x_{i}^{2}\sum_{i}y_{i}^{2}}}\text{.}$$

Average-linkage rule was used for clustering. This algorithm computes a dendrogram that assembles all elements into a single tree. For any set of n genes, an upper-diagonal similarity matrix is computed that contains similarity scores for all pairs of genes. This matrix is scanned to identify the highest value. A node is created to join these two genes, and a gene expression profile is computed for the node by averaging observation for the joined elements. The similarity matrix is updated with the new node replacing the two joined elements, and this process is repeated until only a single element remains [8].

### Identification of differentially expressed genes

The volcano plot method is one of the most widely used method to identify statistically significant differentially expressed genes between two conditions. Each point in volcano plot represents a probe set or a gene, and the x-coordinate represents the (log) fold-change (FC) and y represents the t-statistic or − log10 of the p-value from a *t*-test. The log (FC) is the unstandardized measure of differential expression, but t-statistic is a noise-level-adjusted standardized measure [9]. In the current reanalysis *t*-test was used, p-value computation type is asymptotic. p-Value is calculated as follows:

$$\begin{array}{l} p‐\mathrm{value}=P\left(X_{1}^{2}>t_{obs}\right)\\ \;=1-2P\left(0<N\leq \sqrt{t_{obs}}\right) \end{array}$$

where X_1_^2^ is chi-square distribution with one degree of freedom. N is standard normal distributed value of variable.

### Statistical analysis of differentially expressed genes

Unsupervised hierarchical clustering of differentially expressed genes shows the relationship among the objects that are represented by a tree whose branch length reflects the degree of similarity between objects. In particular, the hierarchical dendrogram can help visualize the object relationship structure between and within clusters. In current analysis Pearson correlation uncentered algorithm was applied with average linkage rule to identify differentially expressed gene expression patterns.

### Significant biology analysis of differentially expressed genes

GO-Elite software [10] was used to identify a non-redundant set of ontology terms, gene sets and pathways enriched in a specified set of genes or metabolites. GO-Elite software has built in databases for diseases and phenotype ontologies, multiple pathway databases, biomarkers, and transcription factor and microRNA targets. GO-Elite also performs advanced over-representation analysis (ORA) statistics from user gene lists, determines the minimal set of biologically distinct ontology terms and pathways from these results and summarizes these results at multiple levels. GO-Elite computes Z-score, p value and q value for each ontology-term, pathway or gene-set. These scores are used for performing the ORA analysis.

## Results

### Quality control and normalization

**Fig. 1:**
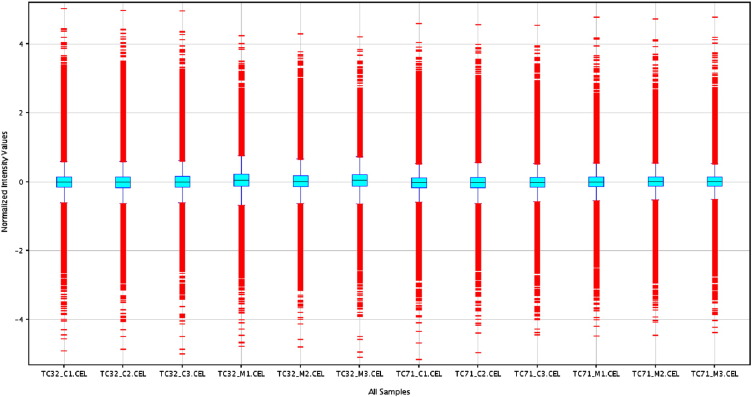
Box plot of Quantile normalized and baseline to median corrected probe expression levels of 12 arrays. The line in the middle of each box represents the median Expression Level in the sample.

**Fig. 2:**
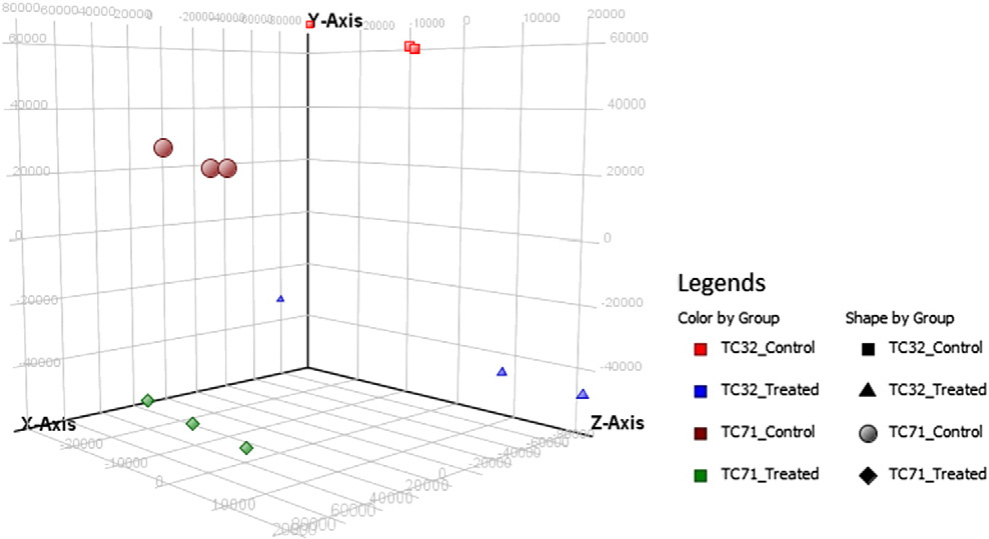
Principal component analysis plot shows one point per array and colored differently based on cell line and experimental conditions.

**Fig. 3:**
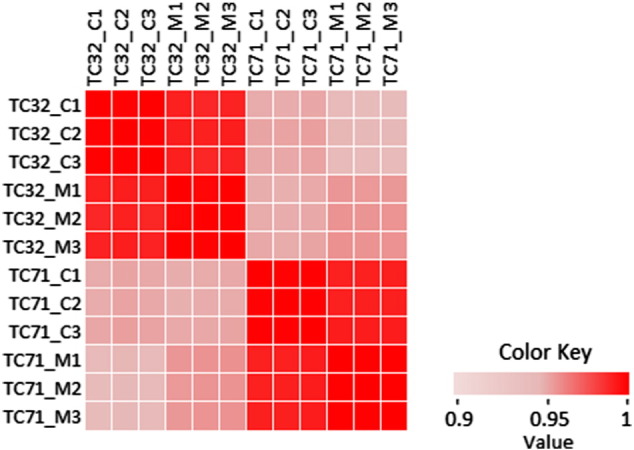
Pearson's Correlation coefficient plot among 12 samples. Probe intensity levels of each array compared at by Pearson's Correlation coefficient indicating strong correlation among the arrays of two cell lines.

**Fig. 4:**
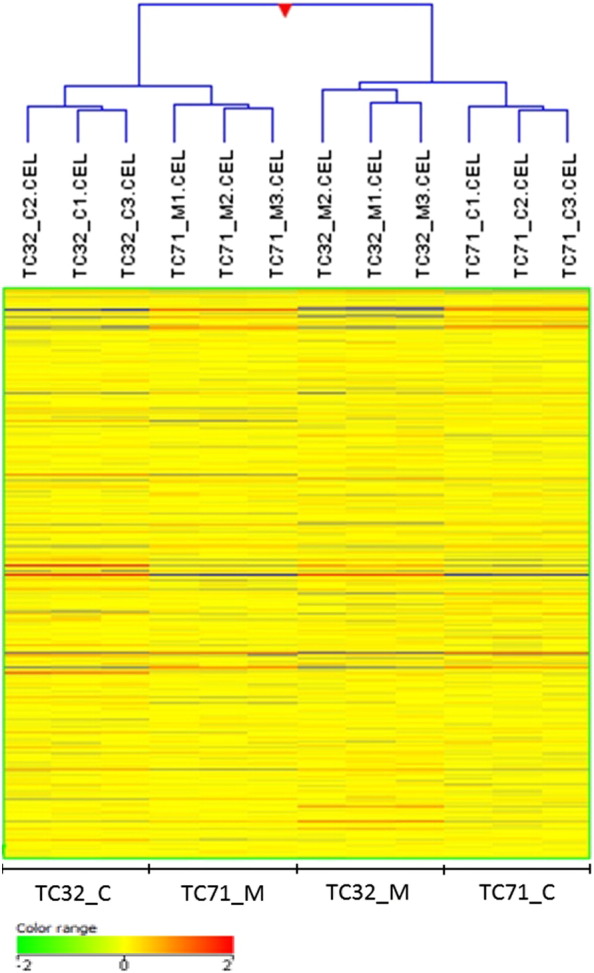
Unsupervised hierarchical cluster analysis on 12 samples of Mithramycin treated and control derived from two cell lines with 3 replicates at each condition. The heatmap shows the expression of 33,469 human genes at the probe level. Heat map colors normalized probe intensity as indicated in the color key. The cluster analysis of mRNA gene expression data separated two cell line specific samples into control and Mithramycin specific samples.

All the 12 samples probe expression values were normalized using Quantile and baseline to median of all the samples. Box Whisker plot analysis (Fig. 1) of the normalized data showed uniform distribution of the expression levels in both intra and intersample manner indicating satisfactory hybridization. Summary statistics showed effectiveness of Quantile normalization as 50th percentile values were close to 0 (Table 1). Principal component analysis (PCA) showed high degree of reproducibility among the replicate samples within each group (Fig. 2). Correlation co-efficient matrix analysis also revealed > 95% correlation between replicate samples (Fig. 3). Further, unsupervised hierarchical condition tree clustered all the replicate samples under the same branch indicating good reproducibility (Fig. 4).

### Differentially expressed genes and cluster analysis

**Fig. 5:**
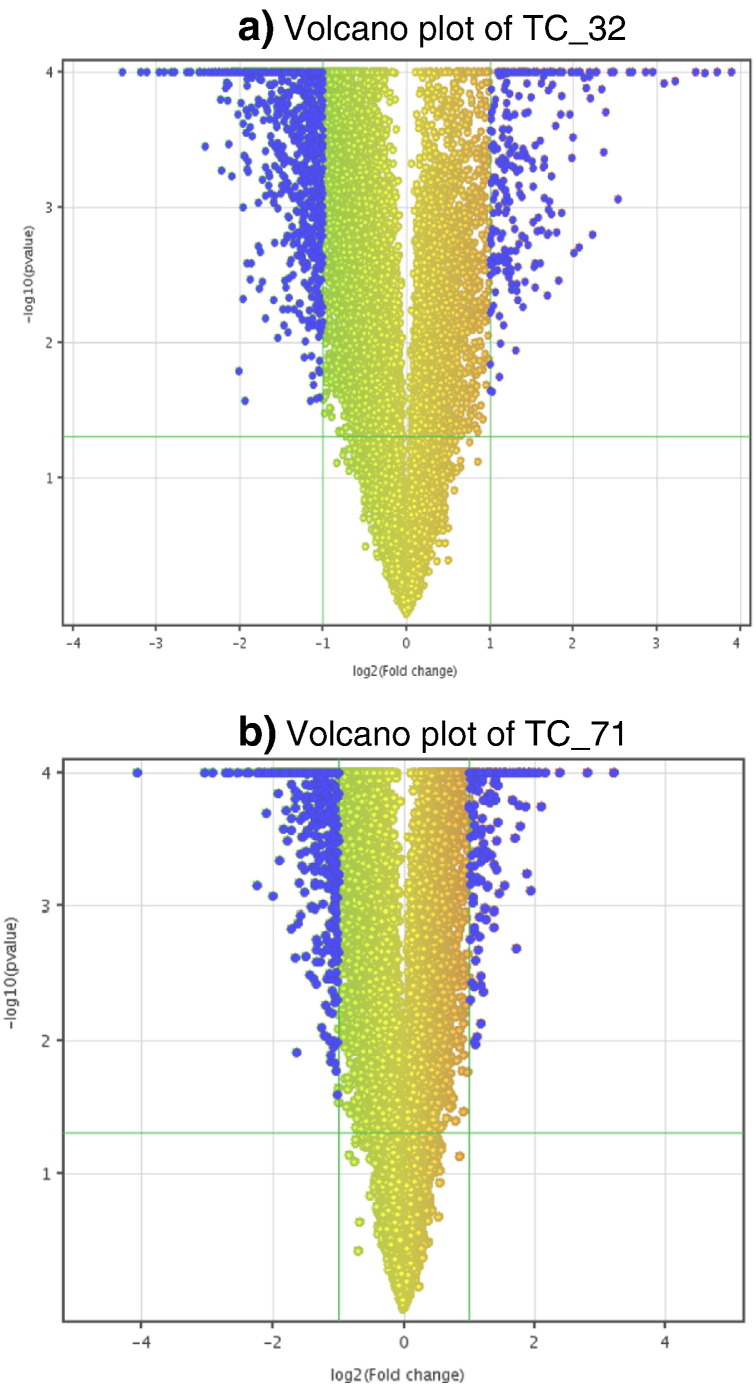
Volcano plot shows distribution of up and down regulated genes with X axis indicating the fold change and Y axis indicating the − log10 p value. Blue highlighted region shows 2 fold up and down regulated genes with p value < = 0.05.

**Fig. 6:**
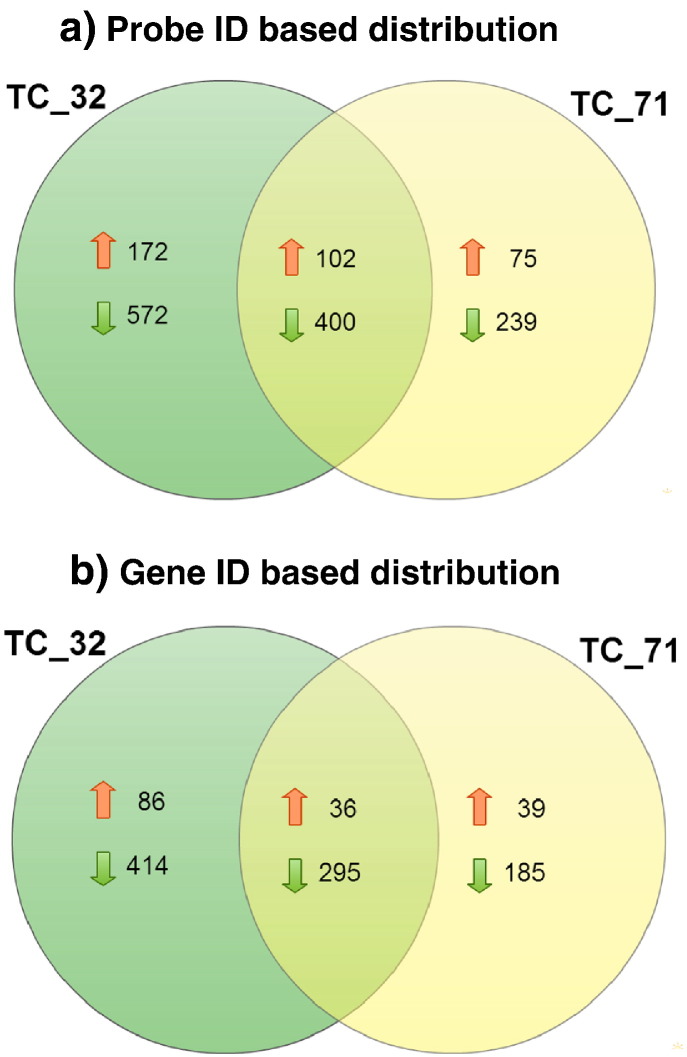
Differential expressed genes across the cell lines TC_32 and TC_71 (Based on Probe IDs and Gene IDs). Up headed arrow indicates up regulation while down headed arrow indicates down regulation.

**Fig. 7:**
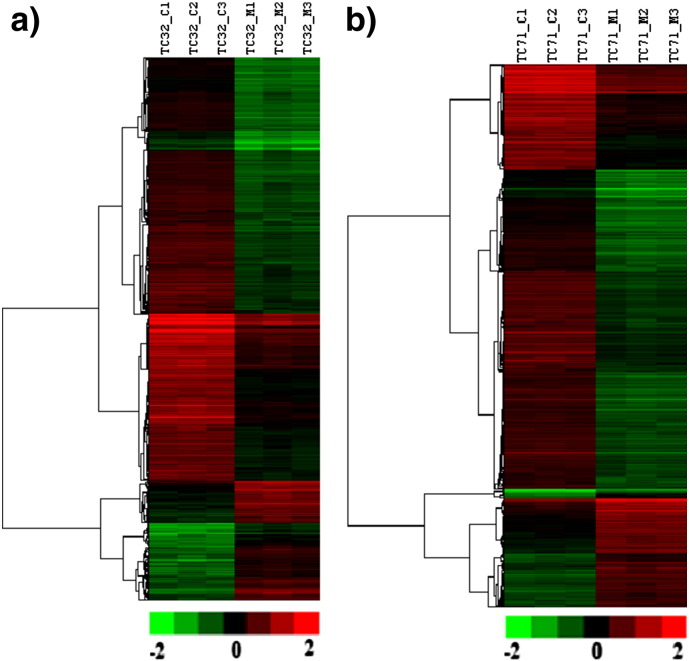
Unsupervised hierarchical clustering of differentially expressed genes shows distinct gene expression patterns upon treatment with reference to the untreated samples.

The volcano plot based method to identify genes that are 2 fold differentially expressed upon treatment in comparison to untreated sarcoma cell lines by applying unpaired Student *t*-test for p-value calculation (p < 0.05) and Benjamini–Hocheberg based FDR correction revealed 1247 genes differentially expressed in TC_32 cell line, while 816 genes were differentially expressed in TC_71 cell line. Down regulation was observed as the prominent regulation in both cell lines [Fig. 5]. Venn diagram representation analysis for understanding distribution of up and down regulated genes across two separate sarcoma cell lines showed probes/genes (based on Entrez Gene ID) that are common and specific to cell line specific manner (Fig. 6). A total of 288 genes were commonly down regulated and 36 genes were commonly up regulated upon treatment in both the sarcoma cell lines. Further, unsupervised hierarchical clustering using Pearson uncentered algorithm with average linkage rule of differentially expressed gene showed distinct patterns of up and down regulated genes upon treatment and also indicates significant reproducibility within the replicate samples (Fig. 7).

### Biological analysis of differentially expressed genes

**Fig. 8:**
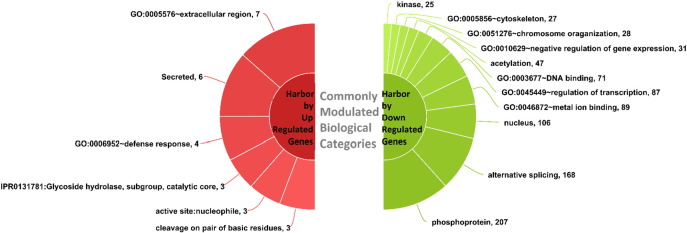
Pie chart representation of significant biological categories that harbor genes down regulated or up regulated by Mithramycin in both sarcoma cell lines generated using high charts [25].

Biological analysis of differentially expressed genes performed using GO-Elite v1.2.5 with over representation analysis (ORA) showed many gene ontology categories, pathways, and protein domains were enriched by differentially expressed genes in both the sarcoma cell lines. The 324 genes that were commonly dysregulated by Mithramycin in both the sarcoma cell lines showed distinct biological categories that were indicative of probable mode of action or effect of Mithramycin on sarcoma cell line. They include, phosphoprotein gene family, genes with metal ion binding capacity & kinases, biological processes like alternative splicing, regulation of transcription, DNA binding, acetylation, negative regulation of gene expression, chromosome organization and cytoskeleton and key cellular component as nucleus for the list of genes that were down regulated. Up regulated genes were mostly secreted and extracellular apart from having role in defense response (Fig. 8).

## Discussion

The role of Mithramycin as an anticancer drug has been well studied [11]. Its antitumor activity is attributed to its GC specific recognition that permits Mithramycin to bind to numerous promoter regions, thereby regulating the expression of downstream genes. Anionic form of Mithramycin has the ability to bind bivalent metal ions and form drug–metal ion complexes which bind to DNA in GC selective manner via the minor groove at and above physiological pH [12], [13]. Thus one of the major intracellular modes of action of this drug is via the association of drug–metal complex with chromosomal DNA in chromatin [14], [15] which results in transcription inhibition. Mithramycin has been found to induce apoptosis by regulating the mTOR/Mcl-1/tsBid pathway in androgen-independent prostate cancer cells [16]. MCL1 is proposed as a key target for Mithramycin A-induced apoptosis in androgen-independent prostate cancer cells and a tumor xenograft animal model [16]. Mithramycin is also reported to repress basal and cigarette smoke-induced expression of ABCG2 and inhibits stem cell signaling in lung and esophageal cancer cells [17]. Histone deacetylase inhibitors and Mithramycin A impact a similar neuroprotective pathway at a crossroad between cancer and neurodegeneration [18]. Modulation of the activity of Sp transcription factors by Mithramycin analogues had shown promising results for treatment of metastatic prostate cancer [19]. Combination therapy using betulinic acid and Mithramycin effectively suppresses pancreatic cancer by inhibiting proliferation, invasion, and angiogenesis [20]. It has been reported that Mithramycin is a gene-selective Sp1 inhibitor that confers a biological intersection between cancer and neurodegeneration [21]. Mithramycin inhibits DNA methyltransferase and metastasis potential of lung cancer cells [22]. Trial of the clinical use of Mithramycin in treating testicular cancer is also well established [23]. Effect of Mithramycin on widespread painful bone metastases in breast cancer is well studied [24]. In this data in “data in brief” article, the effect of Mithramycin in two different sarcoma cell lines was analyzed in a global manner. Global gene expression profiling analysis showed repression of phosphoproteins, kinases, alternative splicing, regulation of transcription, DNA binding, regulation of histone acetylation, negative regulation of gene expression, chromosome organization or chromatin assembly and cytoskeleton.

## Figures and Tables

**Table 1:** Summary statistics.

Property	TC32 C1	TC32 C2	TC32 C3	TC32 M1	TC32 M2	TC32 M3	TC71 C1	TC71 C2	TC71 C3	TC71 M1	TC71 M2	TC71 M3
No. of observations	54,675	54,675	54,675	54,675	54,675	54,675	54,675	54,675	54,675	54,675	54,675	54,675
No. of missing values	0	0	0	0	0	0	0	0	0	0	0	0
Minimum	− 4.90	− 4.85	− 4.99	− 4.77	− 4.78	− 5.09	− 5.14	− 4.95	− 4.43	− 4.46	− 4.45	− 4.37
Maximum	5.05	4.99	4.97	4.24	4.30	4.22	4.61	4.58	4.55	4.78	4.74	4.78
Mean	0.01	0.00	0.01	0.01	0.00	0.01	− 0.01	0.00	0.01	0.01	0.01	0.00
Trimmed mean	0.00	0.00	0.01	0.01	0.00	0.01	− 0.01	0.00	0.00	0.01	0.01	0.01
Median	0.00	− 0.02	0.00	0.05	0.01	0.04	− 0.02	− 0.03	− 0.02	0.00	0.01	0.00
Std. deviation	0.47	0.47	0.47	0.45	0.44	0.44	0.42	0.43	0.42	0.41	0.41	0.41
Trimmed std. deviation	0.36	0.36	0.36	0.36	0.34	0.35	0.32	0.33	0.31	0.32	0.31	0.31
No. of outliers	**6551**	**6287**	**6420**	**4667**	**5172**	**4695**	**5918**	**5482**	**5806**	**6137**	**6223**	**6661**
Percentile 1.0	− 1.41	− 1.38	− 1.36	− 1.46	− 1.36	− 1.41	− 1.17	− 1.12	− 1.13	− 1.24	− 1.30	− 1.28
Percentile 5.0	− 0.66	− 0.63	− 0.65	− 0.76	− 0.66	− 0.72	− 0.52	− 0.50	− 0.49	− 0.55	− 0.61	− 0.58
Percentile 10.0	− 0.41	− 0.40	− 0.40	− 0.49	− 0.41	− 0.46	− 0.35	− 0.34	− 0.32	− 0.34	− 0.36	− 0.35
Percentile 25.0	− 0.15	− 0.17	− 0.15	− 0.13	− 0.15	− 0.13	− 0.17	− 0.18	− 0.16	− 0.14	− 0.12	− 0.12
Percentile 50.0	**0.00**	**− 0.02**	**0.00**	**0.05**	**0.01**	**0.04**	**− 0.02**	**− 0.03**	**− 0.02**	**0.00**	**0.01**	**0.00**
Percentile 75.0	0.15	0.14	0.15	0.23	0.18	0.21	0.11	0.12	0.12	0.14	0.15	0.14
Percentile 90.0	0.42	0.43	0.42	0.41	0.39	0.39	0.39	0.43	0.40	0.39	0.35	0.36
Percentile 95.0	0.70	0.71	0.69	0.55	0.59	0.53	0.64	0.69	0.66	0.62	0.56	0.59
Percentile 99.0	1.57	1.60	1.54	1.14	1.25	1.14	1.42	1.45	1.40	1.32	1.24	1.28

50th percentile value of ± 0.2 from 0 is indicative of effective normalization and good quality hybridization. All the samples in the analysis showed the values within the allowed range and hence it was bold.

## Appendix A Supplementary data

Supplementary Table 1 Absolute expression levels and Fold change of all the 33469 unique transcripts measured in both the sarcoma cell line before and after Mithramycin treatment.

Supplementary Table 2 ORA (Over Representation Analysis) output of differentially expressed genes in two different sarcoma cell lines performed using GOElite tool.
